# Time-Dependent Toxic and Genotoxic Effects of Zinc Oxide Nanoparticles after Long-Term and Repetitive Exposure to Human Mesenchymal Stem Cells

**DOI:** 10.3390/ijerph14121590

**Published:** 2017-12-18

**Authors:** Pascal Ickrath, Martin Wagner, Agmal Scherzad, Thomas Gehrke, Marc Burghartz, Rudolf Hagen, Katrin Radeloff, Norbert Kleinsasser, Stephan Hackenberg

**Affiliations:** 1 Department of Otorhinolaryngology, Plastic, Aesthetic and Reconstructive Head and Neck Surgery, University of Wuerzburg, 97080 Würzburg, Germany; wagnermartin924@gmail.com (M.W.); scherzad_a@ukw.de (A.S.); gehrke_t@ukw.de (T.G.); hagen_r@ukw.de (R.H.); radeloff_k@ukw.de (K.R.); hackenberg_s@ukw.de (S.H.); 2 Department of Otorhinolaryngology, Head and Neck Surgery, Katharinenhospital Stuttgart, 70174 Stuttgart, Germany; m.burghartz@klinikum-stuttgart.de; 3 Department of Otorhinolaryngology, Head and Neck Surgery, Kepler University Hospital, 4021 Linz, Austria; Norbert.Kleinsasser@kepleruniklinikum.at

**Keywords:** zinc oxide, ZnO, nanoparticles, cytotoxicity, toxicity, genotoxicity

## Abstract

Zinc oxide nanoparticles (ZnO-NP) are widely spread in consumer products. Data about the toxicological characteristics of ZnO-NP is still under controversial discussion. The human skin is the most important organ concerning ZnO-NP exposure. Intact skin was demonstrated to be a sufficient barrier against NPs; however, defect skin may allow NP contact to proliferating cells. Within these cells, stem cells are the most important toxicological target for NPs. The aim of this study was to evaluate the genotoxic and cytotoxic effects of ZnO-NP at low-dose concentrations after long-term and repetitive exposure to human mesenchymal stem cells (hMSC). Cytotoxic effects of ZnO-NP were measured by the 3-(4,5-dimethylthiazol-2-yl)-2,5-diphenyl tetrazolium bromide (MTT) assay. Furthermore, genotoxicity was evaluated by the comet assay. For long-term observation over 6 weeks, transmission electron microscopy (TEM) was applied. The results of the study indicated cytotoxic effects of ZnO-NP beginning at high concentrations of 50 μg/mL and genotoxic effects in hMSC exposed to 1 and 10 μg/mL ZnO-NP. Repetitive exposure enhanced cyto- but not genotoxicity. Intracellular NP accumulation was observed up to 6 weeks. The results suggest cytotoxic and genotoxic potential of ZnO-NP. Even low doses of ZnO-NP may induce toxic effects as a result of repetitive exposure and long-term cellular accumulation. This data should be considered before using ZnO-NP on damaged skin.

## 1. Introduction

Nanoparticles (NP) are commercially widely spread. Due to their specific physical and chemical characteristics, they are increasingly gaining importance for biological and biomedical applications [1,2,3]. However, the toxicology of NP is still under controversial discussion [3]. Among the wide range of metal oxide NP, titanium dioxide (TiO_2_) and zinc oxide (ZnO) belong to the most commonly used materials, especially in manufacturing paints and in the cosmetic industry [4]. Previous studies have shown different genotoxic and cytotoxic risk potential of ZnO-NP in several cell lines as well as in primary cells. Although some authors declared ZnO-NP to be without any toxic effects [5], there are numerous studies demonstrating genotoxic and cytotoxic effects of ZnO-NP, even at concentrations which could be relevant for workers in the chemical industry or even for consumers [6]. A previous study of our own group identified ZnO-NP as potentially geno- and cytotoxic, since DNA damage and cell death were induced in human nasal mucosa cells at concentrations of 10 and 50 μg/mL, respectively [7].

Most studies regarding NP toxicology include short-term exposure experiments. Thus, they address acute toxic effects of nanomaterials. However, long-term or repetitive exposure studies are rare. Primary human mesenchymal stem cells (hMSC) can be cultured over several passages, although they are neither transformed nor immortalized. Thus, they represent an excellent non-malignant target tissue for long-term cell culture investigations [8]. Additionally, they are discussed in the current literature as possible vehicles for therapeutic NP in oncological settings [9]. Our study group has previously demonstrated cytotoxic effects of ZnO-NP in hMSC [10]. Furthermore, a particle accumulation in the cytoplasm was observed over a cultivation period of three weeks. However, the tested NP concentrations were higher than in consumer products. The question arises as to whether ZnO-NP at low, non-toxic concentrations will persist in the cytoplasm of hMSC over a longer period of time in an in vitro setting. Consequently, repetitive exposure of non-toxic amounts of NP could lead to critical intracellular concentrations via particle accumulation. The aim of the current study was to evaluate time-dependent cyto- and genotoxicity of ZnO-NP in primary hMSC even in long-term settings and to evaluate the impact of repetitive exposures. Finally, ZnO-NP toxicity was assessed for dependency on short-term differentiation of MSC into adipogenic and osteogenic lineages.

## 2. Materials and Methods

### 2.1. Chemicals

ZnO-NP (<100 nm, purity 99.6%, specific surface area 15–25 m^2^/g) were purchased from Sigma-Aldrich (St. Louis, MO, USA). A quantity of 10 mg of ZnO-NP was suspended in 870 μL distilled aqua and sonicated (Bandelin, Sonopuls HD 60, Berlin, Germany) for 120 s at 4.2 × 10^5^ kJ/m^3^ using a continuous mode to create a high grade of dispersion. According to a protocol by Bihari et al. [11], and modified by Koch et al. [12], 30 μL of 1.5 mg/mL bovine serum albumin (BSA) was added in order to stabilize the dispersion. Finally, 100 μL of 10× concentrated phosphate buffered saline (PBS) was used to achieve a physiological salt concentration and pH 7.4. This stock suspension (10 mg/mL) was subsequently diluted with Bronchial Epithelial Cell Growth Medium (BEGM).

The size distribution of particle aggregates and the zeta potential were evaluated by dynamic light scattering (Malvern Instruments Ltd., Herrenberg, Germany). Particle sedimentation in the cell culture medium was determined by absorption measurements. After sonication, a dispersion of 10 µg/mL ZnO-NP was prepared. The absorbance at 490 nm of the withdrawn aliquots was measured using a TECAN Multimode-Microplate-Reader Infinite^®^ M1000 PRO (Tecan Group Ltd., Männedorf, Switzerland). As a reference, the pure medium with supplements was measured at the four time points (0 h, 4 h, 8 h, and 12 h). We assessed the percentage of non-sedimented NP, defining the measurement at 0 h as 100% and the cell culture medium without NP as 0% [13]. Characterization of shape and single particle size was performed by transmission electron microscopy (see Section 2.7). To analyze the impact of dissolved zinc ions in the medium, ion concentrations were measured. Therefore, RPMI 1640 medium was supplemented with 10 μg/mL ZnO-NP and centrifuged after one hour. Soluble Zn^2+^ ions in the supernatant culture medium were measured by atomic absorption spectrometry (SpectraAA 55 B, Varian GmbH, Darmstadt, Germany). Time-dependent measurements were performed after 1, 3, 6, 12, 24, and 48 h.

### 2.2. Isolation and Culture of Human Adipose Tissue-Derived Mesenchymal Stem Cells

Human adipose tissue-derived MSC were isolated from the subcutaneous adipose tissue of 8 healthy donors. The studies were approved by the local Ethics Committee (12/06), and informed consent was obtained from all individuals included in the study. The isolation procedure was performed according to the methods described by Lee et al. [14] and as previously shown [14,15]. Briefly, adipose tissue was extensively washed with PBS and enzymatically digested at 37 °C for 3 h at a concentration of 15 mg collagenase P (Roche Diagnostics GmbH, Mannheim, Germany) per 100 mL lipoaspirate. To obtain the cell pellet, three washing and centrifugation steps were performed. The pellet was re-suspended and passed through a 100 μm cell strainer (BD Bioscience, Heidelberg, Germany) to remove debris. Cells were plated in culture flasks at 37 °C/5% CO_2_. The expansion medium (DMEM-EM) consisted of Dulbecco’s Modified Eagle Medium, 10% fetal calf serum (FCS) (Linaris, Wertheim-Bettingen, Germany), and 1% penicillin/streptomycin (Sigma-Aldrich, St. Louis, MO, USA). After 24 h, hMSC were rinsed with PBS to remove residual non-adherent cells. The medium was changed every other day. When cells reached >70% confluence, they were detached with 0.25% trypsin (Gibco Invitrogen, Waltham, MA, USA), re-suspended in DMEM-EM and subcultured at a concentration of 2000 cells/cm^2^. Cell morphology was analyzed by microscopy (Leica DMI 4000B Inverted Microscope, Leica Microsystems, Wetzlar, Germany).

### 2.3. Cytotoxicity Evaluation of ZnO-NP

Cells were seeded at a density of 1 × 10^4^ per well in 96-well round bottom plates. Cell culture exposure of hMSC to ZnO-NP was performed at final concentrations of 0.01, 0.1, 1, 10, and 50 μg/mL for 24 h. A quantity of 200 μM tert-butylhydroperoxide (t-BHP) served as the positive control, and DMEM-EM without ZnO-NP as the negative control. After 24 h of exposure at 37 °C and 5% CO_2_ cells were washed twice in DMEM-EM and the 3-(4,5-dimethylthiazol-2-yl)-2,5-diphenyl tetrazolium bromide (MTT) colorimetric staining method [16,17] was used to assess cell viability [18]. All plates were incubated with DMEM-EM containing 1 mg/mL of MTT (Sigma-Aldrich, St. Louis, MO, USA). After 4 h of inoculation, the MTT solution was replaced by isopropanol at 37 °C with 5% CO_2_. Isopropanol was allowed to solubilize the resulting formazan crystals overnight at 37 °C with 5% CO_2_. The color conversion of the blue formazan dye was measured by a spectrophotometer (Titertek Multiskan PLUS MK II, Labsystems, Helsinki, Finland) at a wavelength of 570 nm. All cytotoxicity studies were carried out in eight independent experiments. As an additional negative control, non-cellular controls were carried out in order to prove freedom of particle interference with the test system.

### 2.4. Genotoxicity Evaluation

The alkaline single-cell microgel electrophoresis (comet) assay was applied to detect DNA strand breaks and alkali labile as well as incomplete excision repair sites in single cells [19,20]. A quantity of 2 × 10^4^ hMSC per well was cultured for 24 h at 37 °C with 5% CO_2_. Afterwards, cell culture exposure to ZnO-NP was performed at final concentrations of 0.01, 0.1, 1, and 10 μg/mL. Particles were removed after 24 h by three washing steps followed by the comet assay. In each genotoxicity assay, 300 µM of directly alkylating methyl methan sulphonate (MMS, Sigma-Aldrich, St. Louis, MO, USA) served as a positive control of genotoxicity without cytotoxic effects. DMEM-EM without ZnO-NP was used as the negative control. The comet assay was performed as previously described [21]. The evaluation of the slides was done on a DMLB fluorescence microscope (Leica Microsystems, Wetzlar, Germany) with a filter system incorporating a green excitation filter (515–560 nm bandpass), a dichromatic beam splitter (580 nm longpass), and an emission filter (590 nm longpass) at a magnification of 400×. For every sample, two slides with 50 randomly selected cells each were counted (total of 100 cells per sample). For analysis of the DNA breaks the COMET 5.5 image system (Kinetic Imaging, Liverpool, UK) was used. The following parameters were analyzed to quantify the induced DNA damage: % DNA in tail (TD), tail length (TL), and Olive tail moment (OTM) as a product of the median migration distance and the percentage of DNA in the tail [22]. In the present study, the percentage of DNA in the tail was applied for statistical analysis. Each experiment was performed in eight patients (eight biological replicates).

### 2.5. Repetitive Exposure to ZnO-NP

All genotoxicity and cytotoxicity assays were also performed with repetitive exposure to non-toxic concentrations of ZnO-NP. Cell culture exposure to ZnO-NP was performed at final concentrations of 0.01, 0.1, 1, and 10 μg/mL for 1 h. After a recovery time of 1 h, the exposure was repeated for a total of three times. The cells were washed extensively between the exposures to ensure that no particles were left on the membrane. The medium was changed and replaced with fresh medium without any particles. NP exposure took place only once in the beginning of the experiment. Prior to every medium change, cells were properly washed three times with PBS until no residual NP were macroscopically visible in the wells. Microscopic control after medium exchange revealed persisting intracellular particles but no free NP in the cell culture medium. Additionally, no free particles were observed during daily light microscopy controls. Afterwards, the MTT and comet assays were performed as described above. For the comet assay, only non-cytotoxic concentrations were used. Thus, results of the cytotoxicity assay after repetitive exposure were necessary to define the concentration range for the genotoxicity test.

### 2.6. Dependency of ZnO-NP-Induced Cytotoxicity on Early Differentiation of hMSC

Adipogenic and osteogenic differentiation was performed as described by Pittenger et al. [23] to evaluate differences in ZnO-NP toxicity in hMSC. Cell culture was performed in a 6-well round bottom plate with 2 × 10^4^ hMSC per well. Adipogenic differentiation was induced by DMEM-EM, 10^−7^ M dexamethasone (Sigma-Aldrich, St. Louis, MO, USA), and 10 mg/mL recombinant human insulin (Sigma-Aldrich, St. Louis, MO, USA). The osteogenic induction medium was composed of DMEM-EM, 10^−7^ M dexamethasone, 10^−3^ M β-glycerophosphate and 2^−4^ M ascorbate-2-phosphate (Sigma-Aldrich, St. Louis, MO, USA). Every third day, the medium was renewed, and differentiation was performed for only 1 week. Afterwards, hMSC were incubated for 24 h with ZnO-NP in concentrations of 0.01, 0.1, 1, and 10 μg/mL and t-BHP as positive control. The MTT test was performed as described above.

### 2.7. Long-Term Exposure and Transmission Electron Microscopy (TEM) Evaluation

Cellular uptake and intracellular accumulation of ZnO-NP were evaluated by transmission electron microscopy (TEM) at multiple points in time, beginning early (after 30 min) and continuing up to long-term exposure (6 weeks). The hMSC were treated with ZnO-NP with a concentration of 10 μg/mL for 0.5 h, 1 h, 1.5 h, 1 day, 1 week, 3 weeks, and 6 weeks. Medium change was performed three times a week. Next, cells were trypsinized and centrifugated at 1300 U/min for 4 min. The pellets were fixed in a fresh solution of 0.1 M sodium cacodylate buffer containing 2.5% glutaraldehyde and 2% formaldehyde. Another fixation followed for 2 h at 4 °C with 2% osmium tetroxide in 50 mM sodium cacodylate (pH 7.2). Subsequently, an overnight staining with 0.5% aqueous uranyl acetate was performed. Specimens were dehydrated, embedded in Epon 812, sectioned into ultrathin slices and examined by TEM (Carl Zeiss, Oberkochen, Germany). The photographic negatives were digitalized by scanning and processed using Adobe Photoshop (Adobe Systems Software Ireland Limited, Dublin, Republic of Ireland).

### 2.8. Statistical Analysis

Statistical analysis was performed using GraphPad Prism Software 6.0c (GraphPad Software Inc., La Jolla, CA, USA). The Wilcoxon Test was used to evaluate statistical significance between the mean cell viability of treated samples compared with the negative control (MTT assay) and mean values of the % DNA in the tail of treatment groups and controls, which were defined as 100%. Differences were considered statistically significant when the *p*-value was less than 0.05.

## 3. Results

### 3.1. Particle Characterization

Particles were spherically shaped, as observed using TEM, with a mean diameter of 55 nm. The mean diameter of the aggregates in the culture medium was 120.68 nm with a polydispersity index (PDI) of 0.136. A PDI smaller than 0.2 indicates a monodisperse dispersion. The zeta potential for the ZnO-NP suspension was −11.2 mV. The percentage of non-sedimented NP in the supernatant was 100% at the beginning of the analysis (0 h), 80% after 4 h, 69% after 8 h, and 56% after 12 h. To analyze the impact of dissolved zinc ions in the medium, ion concentrations were measured. Time-dependent measurements of the dissolution of Zn^2+^ ions in the medium were performed in order to clarify if the observed effects are influenced by rising concentrations of ions. Results indicate that ions dissolve shortly after application to the medium. After 1, 3, and 6 h, a concentration of 170 μmol/L; after 12 h, 200 μmol/L; 24 h, 210 μmol/L; and after 48 h, 240 μmol/L zinc ions were measured. Exposure to the particle-free supernatants containing zinc ions did not induce toxic effects. Intracellular Zn^2+^ ion concentration was not measured. However, the authors claim that these intracellular ions are relevant for cell damage.

### 3.2. Cytotoxicity Evaluation of ZnO-NP after 24 h and Repetitive Exposure

**Figure 1:**
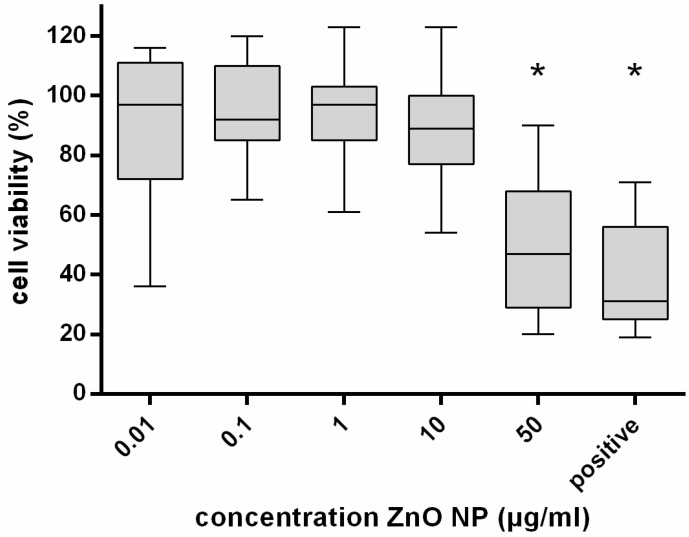
Viability of human mesenchymal stem cells (hMSC) after 24 h exposure to ZnO nanoparticles (NP); *n* = 8. The untreated control was defined as 100%. A quantity of 200 μM tert-butylhydroperoxide (t-BHO) served as positive control. The box plots show the lowest and highest values of viable cells as well as the median. A significant decrease of hMSC viability (%) was observed after exposure to ZnO-NP concentrations of 10 and 50 μg/mL. For statistical analysis, the Wilcoxon Test was performed. *: Statistical significance compared with the control based on a *p*-value < 0.05.

Cell viability after 24 h of ZnO-NP exposure was measured by the MTT assay. Results are expressed in % viability in relation to the untreated control. The MTT test indicated a significant reduction in viable cells dependent on the ZnO-NP concentration, beginning at 50 µg/mL (mean: 48%) in comparison to the control with a *p*-value < 0.05. Results are demonstrated in Figure 1.

**Figure 2:**
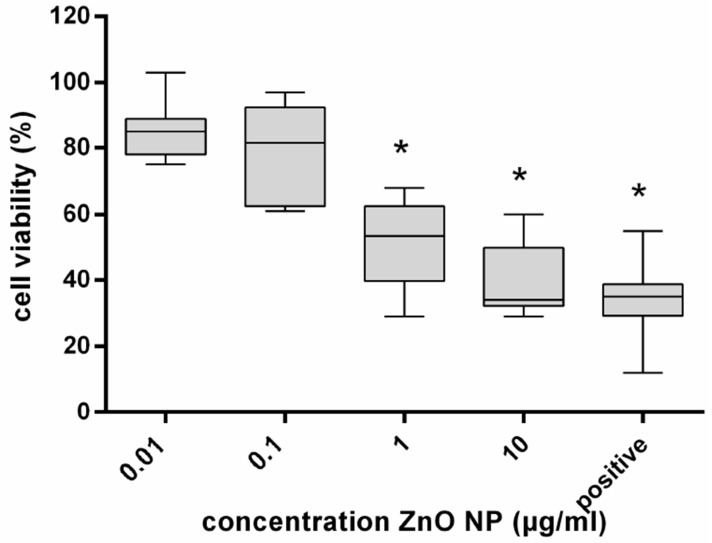
Viability of human mesenchymal stem cells (hMSC) after repetitive exposure to ZnO-NP; *n* = 8. The untreated control was defined as 100%. A quantity of 200 μM tert-butylhydroperoxide (t-BHO) served as positive control. The box plots show the lowest and highest values of viable cells as well as the median. A significant decrease of hMSC viability (%) was observed after exposure to ZnO-NP concentrations of 1 and 10 μg/mL. For statistical analysis, the Wilcoxon Test was performed. *: Statistical significance compared with the control based on a *p*-value < 0.05.

After three repeated exposures of hMSC to ZnO-NP at concentrations from 0.01 µg/mL to 10 µg/mL for one hour, respectively, as described above, there was a significant reduction of cell viability beginning at 1 µg/mL (Figure 2). At 10 µg/mL, there was a mean cell viability of 33.8%. In comparison to a single exposure for 24 h, repetitive exposure led to an intensified decrease in cell viability.

### 3.3. Cytotoxicity in Partially Differentiated hMSC

**Figure 3:**
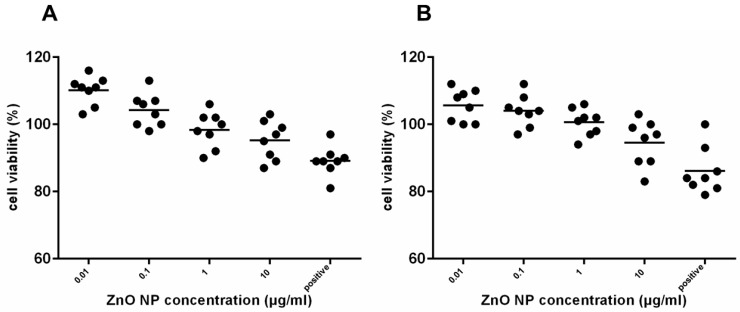
Viability of human mesenchymal stem cells (hMSC) after (**A**) osteogenic and (**B**) adipogenic differentiation for 1 week and exposure to ZnO-NP. Sample size *n* = 8. The untreated control was defined as 100%. A quantity of 200 μM tert-butylhydroperoxide (t-BHO) served as positive control. The dots show the single values of viable cells as well as the median. No significant decrease of hMSC viability (%) was observed after exposure to ZnO-NP concentrations of 0.01, 0.1, 1, and 10 μg/mL. For statistical analysis, the Wilcoxon Test was performed. Statistical significance compared with the control based on a *p*-value < 0.05.

**Figure 4:**
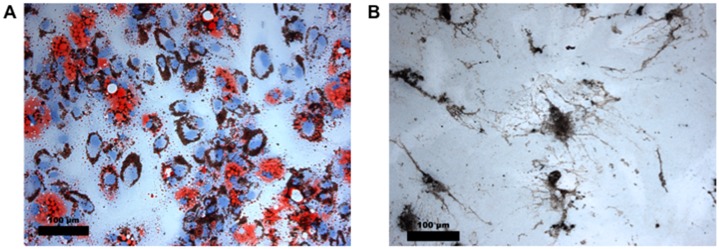
(**A**) hMSC treated with ZnO-NP for 24 h and cultured in adipogenic medium for 1 week. Dark areas in the cell cytoplasm represent ZnO-NP. The adipogenic differentiation was confirmed by staining with Oil Red O to show the presence of intracellular lipid droplets (scale bar representing 100 µm). (**B**) Microscopic analysis of hMSC cultured in osteogenic medium after ZnO-NP treatment. The van Kossa staining characterizes the mineralization by marking the calcium mineral components (scale bar representing 100 µm).

After partial osteogenic differentiation for 1 week and ZnO-NP exposure for 24 h, the MTT assay was performed as described above. No significant cytotoxic effects of ZnO-NP were measured at 0.01, 0.1, 1, and 10 µg/mL ZnO-NP (Figure 3A). After partial adipogenic differentiation for 1 week and ZnO-NP exposure for 24 h, similar results were observed, as no significant reduction of cell viability was seen (Figure 3B). Images are presented in Figure 4.

### 3.4. Genotoxicity Evaluation of ZnO-NP after 24 h and Repetitive Exposure

**Figure 5:**
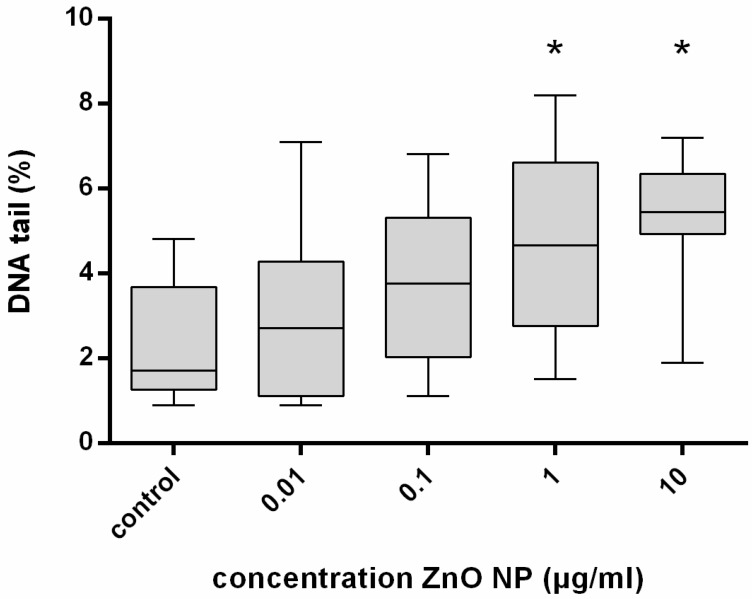
DNA damage expressed by the percentage of DNA in the tail of hMSC after exposure to ZnO-NP. Bronchial Epithelial Cell Growth Medium served as control, and 300 mM MMS as positive control. Cells were treated for 24 h; *n* = 8. For statistical analysis, the Wilcoxon Test was performed. Significant increase in DNA damage was observed at ZnO-NP concentrations of 1 and 10 μg/mL. *: Statistical significance compared with the control based on a *p*-value < 0.05.

There was a significant enhancement of DNA damage, as determined by the comet assay, in hMSC exposed to 1 and 10 µg/mL ZnO-NP for 24 h compared with the control. Cells treated with the positive control, MMS, showed the highest DNA migration in the comet assay (Figure 5).

**Figure 6:**
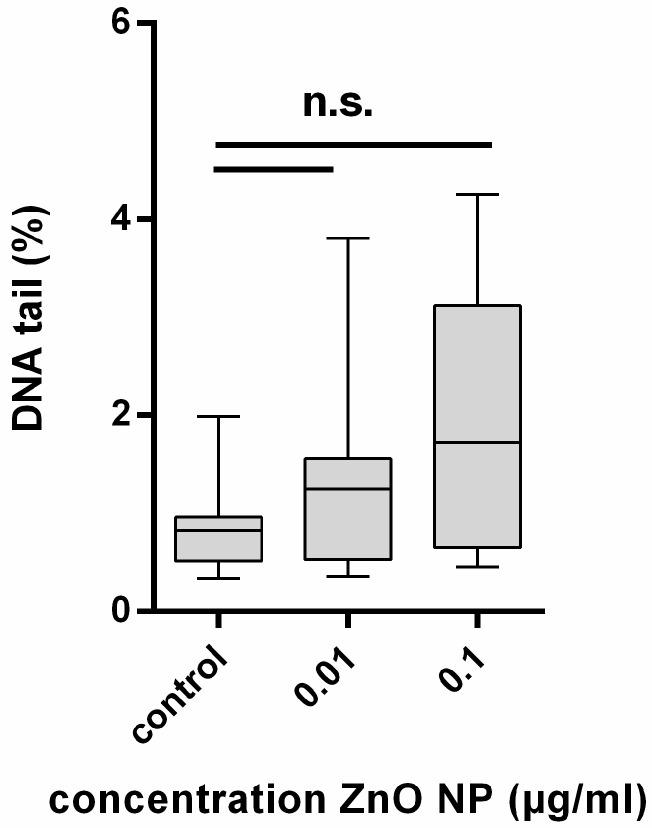
DNA damage expressed by the percentage of DNA in the tail of hMSC after repetitive exposure to ZnO-NP. BEGM served as control, *n* = 8. For statistical analysis, the Wilcoxon Test was performed. Statistical significance compared to the control based on a *p*-value < 0.05. No significant increase in DNA damage was observed. n.s.: none signifanct.

Due to the cytotoxic effects at concentrations of 1 µg/mL and higher after repetitive exposure with ZnO-NP, as described above, measurements of genotoxic effects were indicated for repetitive exposure to 0.01 and 0.1 µg/mL ZnO-NP only. Within these two concentrations, no enhanced DNA damage could be found (Figure 6).

### 3.5. Time-Dependent Cellular Uptake and Accumulation of ZnO-NP in hMSC 

**Figure 7:**
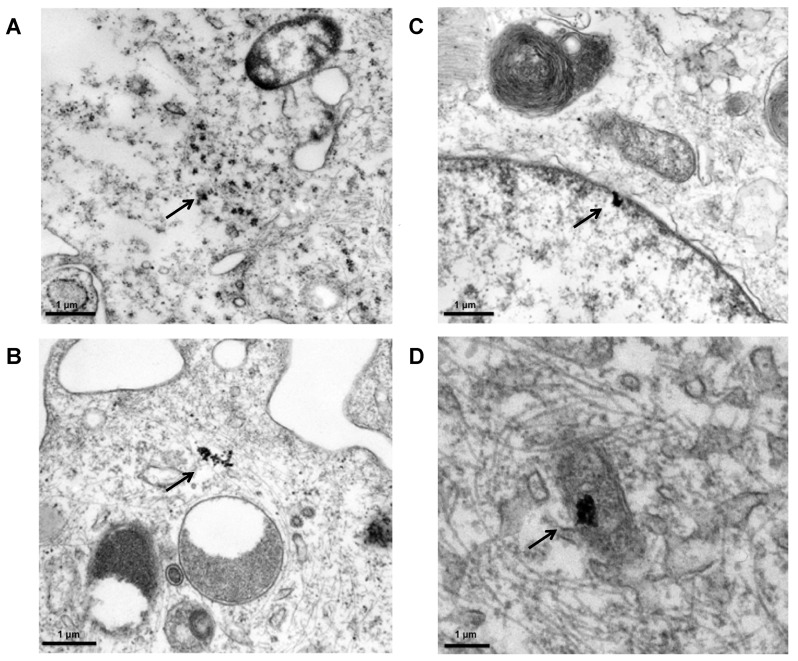
TEM photograph of human mesenchymal stem cells after exposure to ZnO-NP. NP are marked with an arrow. (**A**) After 1 h of exposure, single nanoparticle uptake of dispersed particles and small aggregates was observed. (**B**) After 3 h exposure to ZnO-NP, larger aggregates accumulate, and (**C**) after 1 week, an additional penetration of solitary NP into the cell nucleus was observed. (**D**) Over the observation period up to 3 weeks, an intracellular accumulation in bigger conglomerates and a penetration into other cell organelles (like the mitochondria) by solitary NP or smaller conglomerates was documented. Images were taken at 3000× magnification. Scale bar representing 1 µm.

No NP uptake into the cells was observed after a short-term exposure of 30 min. After 60 min of exposure to ZnO-NP, cellular uptake of dispersed particles and small aggregates was observed (Figure 7A). Intracellular NP accumulation could be described after 180 min of exposure (Figure 7B). After 1 week (Figure 7C), an additional penetration of solitary NP into the cell nucleus and other cell organelles was found. After 3 weeks of exposure (Figure 7D), and over the whole observation period up to 6 weeks, an intracellular accumulation in large conglomerates and a penetration of solitary NP into cell organelles was documented using TEM.

## 4. Discussion

ZnO-NP are one of the most common used nanomaterials. They are applied in medical solutions, cosmetic products, and dietary supplements [24,25]. Due to their UV-protective value, ZnO-NP are ingredients of sunscreens and other cosmetic products. Therefore, the skin is the most important organ which comes into contact with ZnO-NP for consumers, but, especially for employees in chemical industries, the respiratory tract is also exposed to ZnO-NP [26]. An intact dermis is considered to be a sufficient protection of the human organism against NP since they are not able to traverse it and reach the stratum germinativum [27,28,29]. However, lesions, like sunburned skin or other epidermis defects, may allow NP to reach deeper layers of the epidermis or the dermis. There, they are able to get into contact with proliferating cells where they may induce cytotoxicity or DNA damage. Stem cells, which are situated in the basal epithelial layers of the human mucosa and skin, are a critical target for NP. While differentiated keratinocytes will, sooner or later, undergo apoptosis in the stratum corneum, stem cells will persist in the tissue. DNA damage in these persisting cells may affect the daughter cells; thus, MSC have to be classified as a critical target for xenobiotics. That is why the direct influence of ZnO-NP (in particular) on stem cells is coming into focus [9]. Our own studies have shown ZnO-NP to be responsible for cytotoxicity in nasal mucosa cells [30,31] or hMSC after short-term exposure [10]. The cancer-killing effects of ZnO-NP were described to begin at concentrations of 2.5 nM [17]. Although the concentrations of ZnO-NP coming into contact with hMSC in the dermis will be significantly lower, repetitive exposure may promote accumulation of non-critical NP amounts in the cells and lead to genotoxic events. With this background, it was interesting to study the effects of low ZnO-NP concentrations in hMSC after repetitive exposure and in long-term experiments.

First, cytotoxicity thresholds had to be defined for the applied NP in this study. There is a significant decrease in cellular viability at 50 µg/mL, which seems to be quite a high concentration compared with data in the literature. However, stem cells are surely supposed to exhibit different vulnerability to NP compared with keratinocytes or mucosa cells, e.g., DNA damage was observed at lower concentrations. Interestingly, repetitive exposure with only a short recovery time of 1 h induced significantly increased cytotoxic effects starting at a concentration of 1 µg/mL. In real life, repetitive exposure is likely to occur, especially regarding the repeated application of sunscreens with an accumulation of particles. Concerning genotoxic effects, repetitive exposure does not lead to an increase of DNA damage. Long-term evaluations revealed the persistence of NP in the cells; in rare cases, single NP were found in the nucleus. Although the direct transfer of these in vitro results into the in vivo situation is not possible, it must be stated that MSC do not seem to possess efficient mechanisms for NP export [10]. The aim of the experiment was to evaluate repetitive exposure of ZnO-NP at non-toxic concentrations. In our opinion, short repetition intervals seemed to be a relevant exposure pattern. Further studies should differentiate between short and long interval exposure. In the first case, only accumulation effects can be measured. In the second case, repair capacity can also be evaluated. In summary, cell recovery was not examined in this study due to the lack of a recovery time of at least 24 h.

A recent study showed an accumulation of ZnO-NP after topical application within hair follicles of the skin, leading to apoptosis and DNA damage of hair follicle stem cells (HFSCs) [32]. Consistent with these findings, the long-term observations of ZnO-NP in hMSC by TEM in the present study showed an intracellular accumulation and penetration of the nucleus, which may favor an ongoing cytotoxic and genotoxic effect even after six weeks.

After adipogenic and osteogenic differentiation, a decrease of ZnO-NP toxicity was observed. In contrast to hMSC, adipocytes and osteocytes are slowly proliferating and, thus, they may not be as vulnerable to NP as hMSC. These findings are similar to those of another group, who declared ZnO-NP to be toxic even at lower concentrations to adipose-derived mesenchymal stem cells [33]. Although ZnO-NP show lower cytotoxicity to differentiated MSC, they also seem to interfere with stem cell differentiation. In addition, our own group already demonstrated reduced mobility of stem cells after ZnO-NP exposure [10]. To summarize, ZnO-NP influence relevant functional properties of MSC like differentiation and mobility.

To analyze the impact of dissolved zinc ions in the medium, ion concentrations were measured. Results indicate that ions dissolve shortly after application to the medium. Exposure to the particle-free supernatants containing zinc ions did not induce toxic effects. The intracellular Zn^2+^ ion concentration was not measured. However, the authors claim that these intracellular ions are relevant for cell damage. The influence of Zn^2+^ ions is discussed in the current literature [34].

Certainly, the interpretation of the current results is difficult. It is possible that the NP persistence in the cells is a purely in vitro effect. We stated that a damaged integrity of the epidermis may clear the way for NP contact to stem cells, but one has to take into account that sunburned or injured skin will be infiltrated by an inflammatory cell subset. Will NP really get into contact with MSC in such a situation, or will it rather be cleared by inflammatory cells? Perhaps a variation of the local pH value may also influence NP toxicity in inflamed skin. All of these questions cannot be answered by this study. However, our data points to critical characteristics of ZnO-NP in MSC. Rapid uptake combined with slow or insufficient export will lead to high concentrations of particles in the cells. With this knowledge, animal experiments should be designed also using non-toxic NP concentrations with repetitive exposure patterns.

Molecular mechanisms of ZnO-NP-induced toxicity are currently discussed in the literature. Most in vitro assessments demonstrate oxidative DNA damage triggered by dissolved Zn^2+^ ions. Genotoxicological investigations regarding ZnO-NP-induced DNA damage address acute exposure situations. Reactive Oxygen Species (ROS) generation and oxidative DNA damage seem to be a crucial mechanism of ZnO-NP-related geno- and cytotoxicity [35].

## 5. Conclusions

In summary, genotoxic and cytotoxic effects of ZnO-NP to hMSC were demonstrated in long-term and repetitive exposure. A protective effect was seen after one week of MSC differentiation into osteogenic and adipogenic lineages. Observations over a total of six weeks indicate a persisting intracellular accumulation of ZnO-NP and an ongoing toxic effect. This investigation indicates a potential risk in repetitively exposed ZnO-NP to proliferating cells.
